# Sleep impairment as a therapeutic target to treat opioid use disorder

**DOI:** 10.1192/j.eurpsy.2025.375

**Published:** 2025-08-26

**Authors:** L. Li

**Affiliations:** Psychiatry, University of Alabama at Birmingham, Birmingham, United States

## Abstract

**Introduction:**

Opioid use disorder (OUD) is a national public health concern. Craving is a diagnostic criterion and is also implicated in the risk of relapse to opioids. Although sleep impairment is believed to relate with OUD, little research to date has examined the relationship between sleep components and craving in individuals with OUD as well as the moderators in the relationship.

**Objectives:**

This cross-sectional study was designed to address the relationship between sleep disturbance and OUD.

**Methods:**

Individuals with current OUD (n=51) were enrolled. Each participant completed validated scales, including the Pittsburgh Sleep Quality Index for sleep, and Barratt Impulsiveness Scale for impulsivity, and the Opioid Craving Scale for cravings. Correlation and moderation analyses were performed using SPSS, v29.

**Results:**

Total scores of the PSQI were significantly associated with cravings (r=0.44, *p*=0.006). Among sleep components, sleep quality, sleep efficiency, and sleep disturbance were significantly associated with cravings, while four other sleep components were not. Impulsivity moderated the relationship between sleep impairment measured using total score of the PSQI and cravings for participants (*b*=0.15, *p*<.05).

**Conclusions:**

Although preliminary, the findings add to literature on craving, impulsivity, and sleep among individuals with OUD. Sleep impairment and impulsivity may be important targets of a comprehensive, long-term treatment plan for some patients with OUD.

**Disclosure of Interest:**

None Declared

